# Effects of stocking density on growth performance, digestive enzyme activity, biochemical composition, and economic efficiency of a new strain of *Oreochromis niloticus* reared in cage culture system at Sindh Pakistan

**DOI:** 10.1002/fsn3.4238

**Published:** 2024-12-10

**Authors:** Habib Ul Hassan, Amjad Ali, Junaid Wattoo, Muhammad Sohail, Muhammad Ahsan Raza, Syed Adeel Hassan, Roohi Kanwal, Muhammad Kabir, Naseem Rafiq, Salim Manoharadas, Mohammad Rizwan Khan, Takaomi Arai

**Affiliations:** ^1^ Department of Zoology (MRC & RC) University of Karachi Karachi Pakistan; ^2^ Fisheries Development Board Ministry of National Food Security and Research Islamabad Pakistan; ^3^ Center of Excellence in Marine Biology University of Karachi Karachi Pakistan; ^4^ School of Natural and Environmental Sciences Newcastle upon Tyne UK; ^5^ Department of Biology Government Postgraduate College Satellite Town Gujranwala Pakistan; ^6^ Department of Biological Sciences Thal University Bhakkar (University of Sargodha, ex‐Sub‐Campus Bhakkar) Bhakkar Punjab Pakistan; ^7^ Department of Zoology Abdul Wali Khan University Mardan Pakistan; ^8^ Department of Botany and Microbiology, College of Science King Saud University Riyadh Saudi Arabia; ^9^ Department of Chemistry, College of Science King Saud University Riyadh Saudi Arabia; ^10^ Environmental and Life Sciences Programme, Faculty of Science Universiti Brunei Darussalam Jalan Tungku Link Gadong Brunei Darussalam

**Keywords:** biological performance, economics and cost–benefit analysis, implications for sustainable aquaculture, new strain, *Oreochromis niloticus*, stocking density

## Abstract

The latest strain of *Oreochromis niloticus* is an altered ecological adaptation for sustainable aquaculture and is necessary to sustain stocking density and reduce physiological stress of the new strain. The present study aimed to determine the optimum stocking density, biological performance, and economic efficiency of the Nile tilapia. The 14,000 healthy seeds and uniform weight (40 ± 2.4 g) sex‐reversed *Oreochromis niloticus* were stocked in four cages, which are cage (1) 20 fish/m^3^, cage (2) 30 fish/m^3^, cage (3) 40 fish/m^3^, and cage (4) 50 fish/m^3^. The fish were fed 30% dietary protein and feeding frequency three times per day and the feeding rate adjusted according to the fish body weight. Results showed a significantly higher growth, weight gain, and specific growth rate in Nile tilapia stocking density on cage (1), 20 fish/m^3^ and cage (2), 30 fish/m^3^ compared to cage (3), 40 fish/m^3^ and cage (4), 50 fish/m^3^ (*p* < .05). The survival and growth rate showed an inverse relationship with stocking density. The feed conversion ratio (FCR) is the lowest in cage (1) and cage (2), which is significantly different from those of other cages (*p* < .05). The profit index (%) was significantly higher at low density. There was no significant variation in the HSI, VSI and CF. The digestive enzymes such as lipase and amylase were secreted highly in the cages (3) and (4) but high protease was secreted in cage (1). The lipid, ash, and protein of the whole fish were reduced to a lower density, but the moisture levels in the fish bodies raised significantly (*p* < .05) with increasing stocking density. The phenomenal regression indicates that 25–35 fish/m^3^ are optimum stocking density for Nile tilapia in the cage culture system. The current study has made significant step toward optimizing the stocking density of a new strain and developing cage culture in Pakistan.

## Graphical abstract

This study investigated the optimum stocking density, biological performance, and economic efficiency of the new strain of *Oreochromis niloticus*. The monosex seeds with uniform weight were stocked in cages. The fish were fed 30% crude protein (CP) and feeding frequency (FF) 3d‐^1^ and feeding rate 5% body weight (BW) d‐^1^. Results showed a significantly (*p* < .05) higher growth in cage (2), 30 fish/m^3^ compared to others. According to the polynomial regression, the optimum density for *Oreochromis niloticus* is 25–35 fish/m^3^ for the sustainable cage culture system.

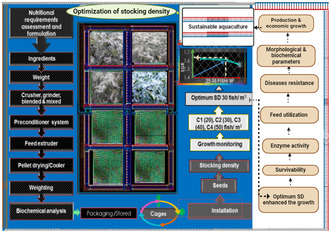

## 1 INTRODUCTION

Aquatic foods are increasingly recognized for their vital role in food security and nutrition, emphasizing the urgent need to use, manage, and sustainably conserve these natural resources (FAO, 2012; Roy et al., 2020). Aquaculture plays a critical role in food and nutritional reliability (Hussain et al., 2021). It is the fastest growing food production technologies in recent decades and decreases overexploitation of wild organisms and prevents ecological decline to provide alternate to the fisherman for their livelihood and to meet the food security demand for the country and globe (Hassan, Ali, Ahmad, et al., 2021). The global population is growing at an alarming rate, with a 30% rise predicted by 2050 (Hassan, Ali, Khan, et al., 2021; Abidin et al., 2022). In response to the increasing demand for fish nutrition and reshaped by sustainable food production opportunities. For the sustainable aquaculture, it is necessary to maximize growth, minimize costs, and promote ecofriendly environmental conditions for excellent economic profit and selection of the quality seed (Hassan, Ali, Ahmad, et al., 2021). The Nile tilapia, the most famous species for aquaculture, is known as aquatic chicken because of its quick growth, strong adaptation to live in difficult ecological condition, and higher disease resistance (Sosa et al., 2005). Due of its taste and meat quality, this species enjoys a high consumer preference. Nile tilapia is well known for aquaculture because of its high market price and great demand (Singh, 2000) and biological advantages (such as fast growth, high food conversion efficiency, short food chain, high disease resistance, breeding in captivity, sexual maturity 5–6 months, and higher fecundity) and physical (tolerance to environmental variability) and social acceptance (nutritional value, taste, and commercial value) (Bwanika et al., 2007; El‐Sayed, 2002; FAO, 2012). Nile tilapia is being cultured globally in open and closed aquaculture systems (Gibtan et al., 2008).

Cage culture is considered as cost–benefit‐intensive form of culture system and one way of gaining production from an area (FAO, 2007; Tsadik & Bart, 2007). It appears as one of the most productive forms of aquaculture and considered as the most excellent way of option for a sustainable aquaculture (eco‐socioeconomically) of several species, including tilapia (Chattopadhyay et al., 2013; Diana et al., 2004; Edwards et al., 2000). The cage culture is a popular choice due to its lower investment compared to costly techniques like raceways and ponds. This method allows for easy growth in water bodies, increasing fish productivity and creating jobs (Baliao & Dosado, 2011; Ridha, 2006; Roy et al., 2020). A cage culture system's financial success depends heavily on figuring out the right fish stocking density for the surrounding environment, given their impact on water quality (FAO, 2012).

Stocking density is one of the crucial factors thought to be crucial to the aquaculture industry's success (Naderi et al., 2017; Zahid et al., 2013). The fish stocking densities directly impact on growth efficiency, survival, health, feed efficiency, productivity, disease control, and environmental contamination prevention, thereby affecting farm profitability (Lin et al., 2004; Ruane et al., 2002). It is a well‐established fact that fish growth rates gradually rise in tandem with decreasing stocking densities (FAO, 2007). On the other hand, overly dense conditions may lead to fish competing with one another for air, food, and space (Abdel‐Aziz et al., 2021). Stocking density takes into account the most effective way to maximize the area unit and boost output (Lin et al., 2004). The maximum production is directly linked to the optimum stocking density (Kapinga et al., 2014). High densities of fish farming can significantly change growth and biological performance in various species (Dong et al., 2018). Conversely, poor stocking densities are less desirable due to poor yield per unit surface area or water volume. Abdel‐Aziz et al. (2021) declared that a stocking density of 100 individuals/1 m^3^ has a negative effect on Nile tilapia growth and biological performances. The stocking density has been reported to be inversely correlated in numerous studies (Chattopadhyay et al., 2013; El‐Sayed, 2002; Lee et al., 2021).

Pakistan is endowed with lots of open water resources like lakes, rivers, dams, creeks and reservoirs, which can be effectively used for genetically improved farmed tilapia (GIFT) production through cage culture. Cage culture technique in Pakistan does not have a long history, thus results are inconsistent and there is not so much encouragement. In Pakistan, cage operators adopt stocking densities and management procedures that are not based on research, leading to poor development and survivability. For the sustainable aquaculture to optimization necessitates the selection of efficient strains. As a result, genetic improvement has emerged as an essential component of aquaculture development. The high stocking density's detrimental impact on the fish growth, FCR and survival and water quality and surrounding environment and declining health of fish and diseases can spread quickly but the low stocking density reduces the production. Earlier study on the stocking density of a non‐genetically improved tilapia was performed, but recently, we investigated the assessment of biological performance, growth, enzyme activity, biochemical composition, and economic efficiency of a new strain of *Oreochromis niloticus* reared under different stocking densities, thus providing an optimum density for cage culture of monosex *O. niloticus* in Pakistan for obtaining a better yield in terms of production and higher profit index in terms of income. To maximize economic returns, the cages would need to be stocked at optimum stocking densities for optimum growth in proportion to inputs and water body productivity for the implications for sustainable aquaculture. The purpose of the study was to look at the effect of stocking density on GIFT innovative strain Nile tilapia culture under the same diet and management circumstances.

## 2 MATERIALS AND METHODS

### 2.1 Ethical approval

The Ethics Committee of the Fisheries Development Board and Department of Zoology (MRCC) approved the experiment.

### 2.2 Experimental site and design

**FIGURE 1:**
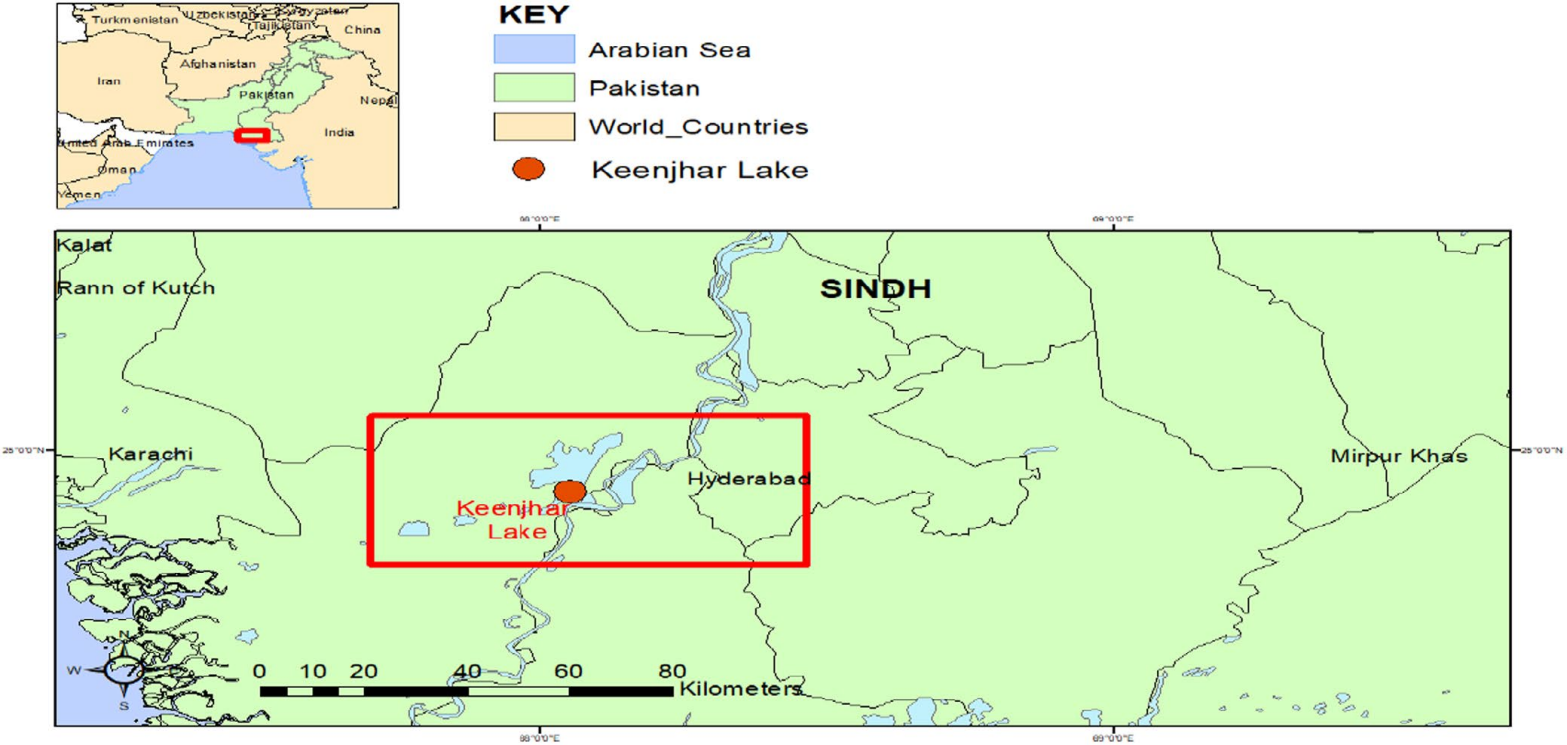
A map indicating the research area of Keenjhar Thatta Sindh, Pakistan.

This research was conducted in the open aquaculture system in Keenjhar Pakistan from May 2023 to October 2023. The cages were installed at Keenjhar lake (N: 24.53′49,470°; E: 68. 3′13,856) (Figure 1).

**FIGURE 2:**
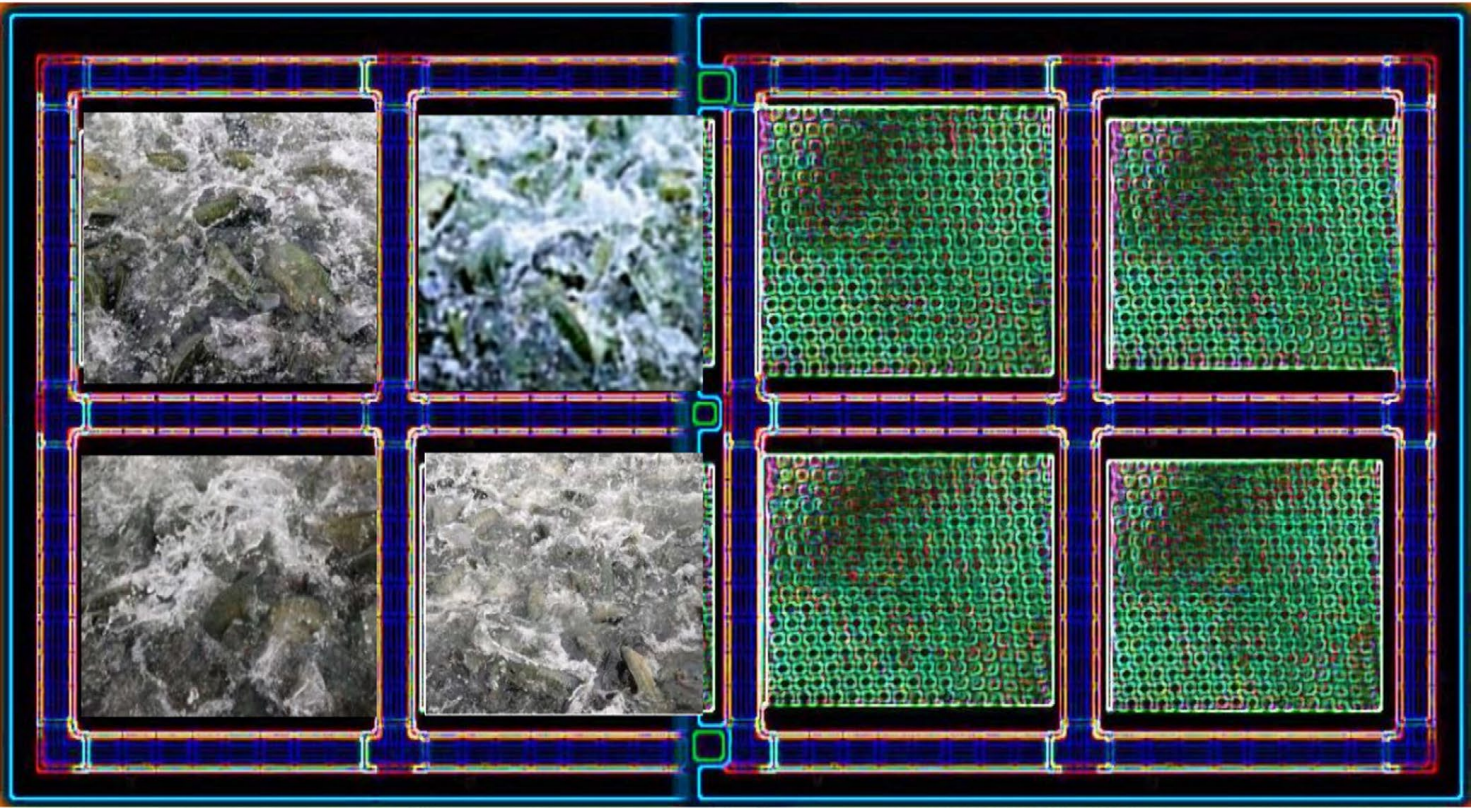
Commercial cage's schematic diagram of one unit/set of ten cages of battery.

The cages were built with knotless plastic net suspended from a quadrangular cage structure made of galvanized iron (GI) pipes set straight above the water level. However, the water level in each cage was maintained consistently by suspending large, floating plastic drums over it. The net's mesh size was maintained at 5 to 6 mm in order to prevent the experimental fish from escaping; these nets are known as grow‐out nets and they are covered by predator control nets. This mesh size, however, was large enough to efficiently move a huge amount of water through the cages. All the cage frames were joined by iron plates, and the whole quadrangular cage structure hung above the water with closed plastic drums placed in between each pair of cages and between the two ends of the cage series, which was also called the battery (Table 1). Human movement (pedestrian) was made possible by a platform made of GI pipes that was placed beside the cage structure in order to give feeds, sample fish, and collect them along the known walkway (Figure 2). During the cultural period, local fishermen participated actively in all activities.

**TABLE 1:** Cage system components and detailed description for one unit/set of ten cages of battery.

Particulars	Description
Unit/Set	Battery of 10 (5 **×** 2) cages and each cage comprising of quick assemble‐able components
Frame	Each cage size: 6 m **×** 6 m inner side: Outer main double pipes: Diameter 50 mm minimum (inner side) high‐quality Gl pipe. Thickness: 11‐gauge—Inner supporting double pipes: Diameter 50 mm minimum (inner side) high‐quality Gl pipe. Thickness: 15/14 gauge minimum
Walkway	Length: All‐around each cage—Width: 30 inches minimum—Meshed (Barfi) mat made of galvanized (GI) 3 mm thickness—Horizontal thickness: 3 mm minimum—Vertical thickness: 5 mm minimum—Mesh size: 1.5 **×** 1.5‐inch maximum—Supporting bars/rods: Cross pipes of 1‐inch diameter on walkway frame were welded at a distance of every 2 ft
Mooring rope	Material: USA tire cut rope (ship anchoring rope)—Thickness: 35–40 mm—Length: 200 m (50 **×** 4 m) or as per the requirement of water depth, whichever is more
Anchor	‐8 Nos. per unit/set weighing 40 kg of each block of reinforced cement concrete (RCC) (1:2:3) with iron hook (1.5‐inch thick min.). ‐80 Nos. (10 **×** 8) per unit/set each weighing 5 kg block of plain cement concrete (PCC) (1:1.5:3) with hook (1/2‐inch‐thick min)
Net holding rope	Thickness: 20–25 mm—Length: 10 **×** 100 m per unit/set (on top, bottom, corner to corner, and side center to center of net)
Drum as a floater	No. of drums per battery: 108 Nos.—Type: Single use double collar hollow plastic drum—Color: Dark blue color—Height/Length: 33 inches—Diameter: 22 inches—Fitting: Fix with GI wire to hold the movement of drum
Cover net (10 Nos.)	Cover net size: 6 m **×** 6 m—Mesh size: 2 inches knot to knot—Thread type: 20 ply polyester
Grow‐out nets (10 Nos.)	‐Net Size: 6 m **×** 6 m **×** 3 m depth including blind net (Length **×** Width **×** Depth)—Quality/Type: high‐density polyethylene (HDPE) net thread 36 ply (Knotless)—Net mesh size: 25–28 mm, 36 ply as per customer choice
Joints	Joints of the main frame were fixed with nuts and bolts and the material made of Gl pipe

The cage was placed in the largest water adjacent to each other having a similar size of (6 × 6 × 3 m) and the total volume per cage was 100 m^3^.The eight cages were selected for rearing out of 10 cages. Two cages for emptying were used during disease outbreaks and growth monitoring. All the cages were washed and kept in sunlight for 10 h and then nets of mesh size 5–6 mm were fixed all‐around each cage.

Sex reversal is one of the characteristics of Nile tilapia sexual plasticity, in which both natural and artificial sex change occurs at various periods of their life cycle. The new strain of Nile tilapia undergoes the all‐female change to male, and the all‐new seeds in the gender identity of an organism from one sex to the opposite (sex reversal) had three times better growth. The Nile tilapia seeds underwent all‐female was change to male through dietary inclusion of hormone and medicinal plant diet and produced the latest seeds and these latest seeds are also called new strain. The seeds of Nile tilapia were collected from a commercial hatchery; prior to packing, fish. Seeds were transported in oxygenated double‐coated polythene bags to lessen their stress. Additionally, ice packs were inserted between the polythene bags' exterior and interior layers to maintain the optimum temperature while the seeds were being transported. The experimental period was preceded by an acclimatization period. After the seeds were placed in the cage, the bags were submerged in water for a period. The seeds' bags was then progressively soaked with water to keep temperature and pH. The 14,000 seeds were distributed into four cages and each treatment had one replicate. The stocking density (SD) of Nile tilapia was distributed in cage 1 (2000), cage 2 (3000), cage 3 (4000), and cage 4 (5000) with an average initial weight of 40 ± 02 g. The stocking density of *O. niloticus* is 20, 30, 40, and 50 fish/m^3^, respectively, and continued rearing for 180 days. The dietary protein level was 30%. The ingredients were purchased from the local market and formulated according to the Hassan et al. (2022). All the ingredients' and biochemical analysis was done and is presented in (Table 2). Initially the feeding rate was 5% and further adjusted according to the fish body weight on a weekly basis and the feeding frequency was three times per day.

**TABLE 2:** Nutritional requirements assessments, composition and biochemical analysis of the experimental diets used as food for the *Oreochromis niloticus* during the rearing period.

Ingredients (%)	Experimental diet ratio
Fish meal	29.0
Soybean	30.0
Wheat flour	14.3
Rice bran	8.0
Bread flour	5.7
CLO	5.0
VP	4.0
MP	2.7
FPH	1.3
Total	100%
Biochemical compositions	
Moisture	12.1 ± 0.1
Crude protein	30 ± 0.0
C‐lipid	6.5 ± 0.5
CF	9.1 ± 0.1
Ash	8.05 ± 0.3
NFE	43.34 ± 0.4

### 2.3 Water quality parameters

Water quality parameters were recorded on a daily basis, such as temperature and pH by Hanna—HI98107, dissolved oxygen (DO) by digital DO‐meter (Model: HI9146) and salinity by refractometer (Model:RHB‐32SG/ATC), while ammonia (mg/L), nitrite‐N, and nitrate‐N were checked every 2 weeks using the American Public Health Association (1995) (Standard Methods for the Examination of Water and Wastewater) spectrophotometric method (Wang et al., 2015).

### 2.4 Whole‐body chemical composition

The experimental diets' biochemical composition (crude protein, crude fat, dietary fiber, ash, and moisture contents) was assessed using the industry standard (AOAC, 2010). Ten fish per group were frozen at a temperature of −20°C, according to the protocols and criteria outlined by the crude protein Kjeldahl technique (N × 6.25). The said technique was utilized to evaluate the crude protein, which involved converting the total nitrogen into crude protein. Using petroleum ether and the Soxhlet extractor, crude lipid was extracted (Hassan et al., 2022).

### 2.5 Calculation of growth parameters

The biotechnical parameters of fish were calculated according to Hassan, Ali, Ahmad, et al. (2021).

ADWG (g) = (FBW) − (IBW) (g))/days, Weight Gain (WG) (g) = FBW (G) − IBW (g); FCR = (Feed consumed (g))/(WG (g)); SGR, %/day = [(W2) − (W1) × 100]/days; Condition factor (CF) (K) = ((BW))/((L^3^)) × 100; Hepatosomatic index (HSI): Liver (g)/empty fish (g) × 100; Viscerosomatic index (VSI) = ((viscera weight))/fish weight × 100; Survival rate (SR %) = (Fish survived)/(Fish released) × 100.

### 2.6 Profitability index

The profitability index is an economic analysis used to find out a profit over investment. It's a simple inquiry to estimate the profitability of each group. Feed cost and fry cost and all expenses incurred during the trial and the total revenue gained from culture were also estimated.

Profit index (PI) = (Total amount of fish yield)/(Total expenses of trial); Incident Cost (IC) = Feed Price/Biomass Yield (kg); Economic conversion ratio (ECR) = feed cost × FCR; Return on Costs (RC) = Profit/Total costs.

### 2.7 Digestive enzyme activity assays

According to Najdegerami et al. (2016) at the end of the research, 10 fish were randomly selected from each cage, put to euthanize with 0.5 mL/L of clove oil, and their whole digestive tract was removed for analysis. After thoroughly cleaning the digestive tract was washed with distilled water and transferred to a sterile tub and homogenized, in pH 7.4 phosphate buffer, the process was repeated (Samanta et al., 2016). The supernatants (enzyme extracts) were extracted after centrifuging the homogenates at 25,000*g* for 20 min at 4°C to remove tissue debris and lipids. They were stored at 4°C, until further examination of digestive enzyme activity. Adineh et al. (2019) and Samanta et al. (2016) described measuring protease activity at 25°C in 0.2 M phosphate buffer at pH 7.0 using 1% (w/v) casein (Sigma, USA) as a substrate (Akter et al., 2016). The proteolytic activity of enzyme extracts was measured using L‐tyrosine as a reference (Adineh et al., 2019) and the amylase activity was measured using starch and carboxymethylcellulose as substrates. Enzyme specific activity was reported as unit enzyme activity per milligram of protein (Clark, 1964).

### 2.8 Statistical analysis

The study utilized SPSS version 22, one‐way analysis of variance (ANOVA), and Duncan's test to analyze data, and the study's goal was to find significant variations in treatment methods at *p* < .05.

## 3 RESULTS

### 3.1 Physiochemical parameters of water

The water quality parameters including DO, temperature, salinity, ammonia, and pH were within an optimal range suitable for the culture of Nile tilapia. While we noticed significant variations in temperature from April to August, there were variations similarly in the dissolved oxygen but within a suitable range for the culture (Table 3).

**TABLE 3:** Stocking density effects on water quality parameter during the rearing period.

Parameters	Stocking density			
	C‐1 (20 fish/m^3^)	C‐2 (30 fish/m^3^)	C‐3 (40 fish/m^3^)	C‐4 (50 fish/m^3^)
Temperature (°C)	28.3 ± 3.8	28.2 ± 3.7	28.2 ± 3.6	28.2 ± 3.5
DO (mg/L)	6.4 ± 0.5	6.2 ± 0.1	5.4 ± 0.7	5.2 ± 0.8
pH	8.4 ± 0.2	8.1 ± 0.1	7.8 ± 0.5	7.4 ± 0.4
Salinity	0.2 ± 0.08	0.2 ± 0.01	0.2 ± 0.1	0.2 ± 0.1
Nitrite (mg/L)	0.01 ± 0.0	0.1 ± 0.0	0.02 ± 0.01	0.02 ± 0.002
Ammonia (mg/L)	0.01 ± 0.0	0.01 ± 0.0	0.02 ± 0.0	0.02 ± 0.0
Nitrate (mg/L)	0.01 ± 0.0	0.1 ± 0.0	0.02 ± 0.02	0.02 ± 0.004

### 3.2 Growth, feed utilization, and morphological indices

**FIGURE 3:**
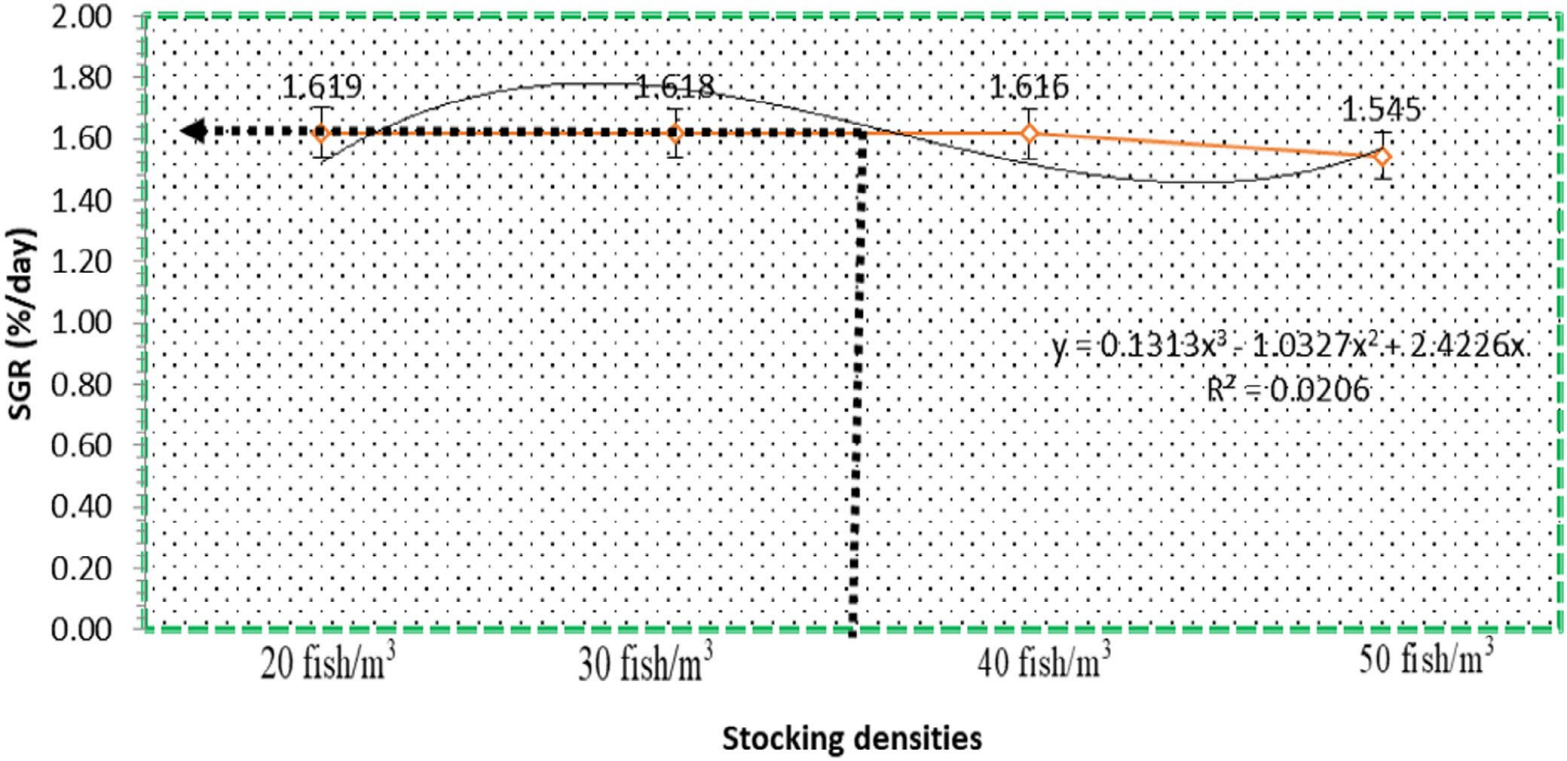
The optimum stocking density is 25–35 fish/m^3^ for Nile tilapia based on SGR% as indicated by the phenomenal regression.

The growth parameter (GP) of *O. niloticus* during the 180 days at different stocking densities is presented in Table 4. Initially, all the weights of fish were non‐significant (*p* > .05). There was a significant difference (*p* < .05) in the GP across the treatments (Table 4). *O. niloticus* in cage (2) increased its WG, final length (FL), average daily weight gain (ADWG), and SGR to the highest levels, followed by cage (1) and cage (3), while cage 4 had the lowest levels of these parameters. The WG and SGR showed an inverse relationship with stocking density, therefore cage (1) group of fish showed higher WG and SGR values as compared to other treatments. While the high stocking densities (fish/cage) of 40 fish/m^3^ and 50 fish/m^3^ have negatively affected the final weight gain as it is significantly high in cage (1) (*p* < .05) (Table 4). Also Figure 3, SGR curving reached the maximum point at SD 25 to 35 fish/m^3^. Increase in SD to decrease in SGR establishing a linear relationship, *y* = 0.1313*x* ^3^ – 1.1327 + 2.4226*x* *r* ^2^ = −.903 (Figure 3). Condition factor (CF) was lower with cage (1) and cage (2) than cage (3) and cage (4), respectively. HSI and VSI of Nile tilapia at cage (3) and (4) were higher than those of the other two treatments of cage (1) and cage (2).

**TABLE 4:** Growth and feed consumption of *Oreochromis niloticus* under different stocking densities.

Attributes	Stocking density			
	C‐1 (20 fish/m^3^)	C‐2 (30 fish/m^3^)	C‐3 (40 fish/m^3^)	C‐4 (50 fish/m^3^)
IBW (g)	40.33 ± 2.33^a^	40.32 ± 2.08^a^	40.32 ± 2.91^a^	40.30 ± 3.46^a^
FBW (g)	744.60 ± 15.96^a^	743.80 ± 10.22^b^	740.75 ± 13.90^c^	650.67 ± 5.60^c^
IBL (cm)	17.71 + 0.22^a^	17.41 + 0.34^a^	17.21 + 0.21^a^	17.64 + 0.21^a^
FBL (cm)	28.42 + 1.64^a^	27.45 + 1.88^a^	25.85 + 1.44^b^	21.85 + 1.44^b^
ADWG (g)	3.91 ± 07.16^a^	3.90 ± 02.95^b^	3.88 ± 0.53^c^	3.38 ± 0.70^d^
WG (g)	704 + 27 ± 08.17^a^	703.48 ± 11.95^b^	700.43 ± 16.43^c^	610.37 ± 4.60^d^
FCR	0.82 + 0.12^a^	0.88 + 0.12^b^	0.94 + 0.12^b^	1.02 + 0.32^b^
SGR (%/day)	2.619 ± 0.7^a^	1.618 ± 0.5^a^	1.616 ± 0.5^b^	1.545 ± 0.3^b^
CF (%)	3.24 ± 0.01^a^	3.59 ± 0.01^a^	4.28 ± 0.02^b^	6.23 ± 0.04^b^
Survival (%)	98 ± 0.00^a^	98 ± 0.00^a^	96 ± 0.00^b^	92 ± 0.00^c^
HSI %	1.3 ± 0.13^a^	1.2 ± 0.33^a^	1.4 ± 0.05^b^	1.4 ± 0.02^b^
VSI %	3.5 ± 0.11^b^	4.5 ± 0.11^a^	4.8 ± 0.21^a^	4.7 ± 0.11^a^

*Note*: The data are displayed as mean standard error. The means of the individual letters inside the columns differ considerably (*p* 0.05). (Tukey's test after ANOVA). Significant difference is represented by an alphabetical superscript.

### 3.3 Survival rate

The survival rate was higher when the SD was decreased to 30 fish per m^3^ than when it was increased to 50 fish per m^3^, the large variations in survival rates observed in treatments; cage (1) and cage (2) had the highest rates, followed by cage (4) with 90% and cage (1) with 96%.

### 3.4 Economic efficiency

Table 5 depicts the influence of SD on economic efficiency's significant differences between the treatments. The cage (2) had the largest profit (1753.87 $), biomass yield (BY) (2229 kg), and profit index (PI) (3.75 $). It also had the best return on costs (RC) (1.87), incident cost (IC) (0.30), and feed cost (FC) (658.29 $), which were much more than those of the other treatments, cage (1), cage (3), and cage (4). The result indicated that higher profit was achieved by stocking fish at lower density as compared to high density. The cage (1) and cage (2) groups showed a handsome profit margin in terms of income than cage (3) and cage (4) groups (Table 5).

**TABLE 5:** Evaluating the economic efficiency of GIFT *Oreochromis niloticus.*

Parameters	Stocking density			
	C‐1 (20 fish/m^3^)	C‐2 (30 fish/m^3^)	C‐3 (40 fish/m^3^)	C‐4 (50 fish/m^3^)
Seed cost (US$)	97.57	146.36	195.14	243.93
Feed cost (US$)	388.99	658.29	927.59	1196.89
Mis. expenses (US$)	130.10	130.10	130.10	130.10
Biomass yield (kg)	1488	2229	2960	3700
Total income (US$)	1693.86	2571.52	2406.79	2406.79
ECR	93.15	105.3	112.05	116.1
Profitability (US$)	1077.20	1753.87	1271.04	952.96
Profit Index	4.35	3.75	2.59	2.01
Incident cost	0.26	0.30	0.31	0.32
Return on costs	1.74	1.87	1.02	0.60

*Note*: Exchange rate: 1 US$ = 290 (= Pakistani Rupees) as on October 01, 2023.

### 3.5 Digestive enzyme activities

The concentrations of enzymatic activities were noted at the end of the trial; considerable variations were noticed with varying concentrations in all the groups. The amylase, protease, and lipase activities were significantly lower (*p* < .05) in the higher density cage (3) and cage (4) compared to cage (1) and cage (2) as shown in Table 6.

**TABLE 6:** Influence of stocking densities on digestive enzyme activities.

Enzymes	Stocking density			
	C‐1 (20 fish/m^3^)	C‐2 (30 fish/m^3^)	C‐3 (40 fish/m^3^)	C‐4 (50 fish/m^3^)
Lipase ((U/mg)prot)	24.85 ± 0.82^a^	23.47 ± 0.65^b^	32.62 ± 0.66^c^	30.75 ± 0.69^d^
Amylase ((U/mg)prot)	0.68 ± 0.02^a^	0.66 ± 0.03^a^	0.58 ± 0.06^a^	0.52 ± 0.05^a^
Protease ((U/mg)prot)	178 ± 6.87^a^	166.46 ± 5.26^b^	140.49 ± 6.92^c^	126.27 ± 4.71^d^

*Note*: The concentrations of enzymes were measured at the end of trial and all the values are presented as mean ± SE. Alphabetical superscript represents significant difference.

### 3.6 Whole‐body composition

In Table 7, the carcass composition of Nile tilapia high in varying SDs is displayed. Between treatments, there were no discernible changes in the tilapia crude lipid or whole‐body composition (*p* > .05). Still, the fish group with the highest SD had significantly lower (*p* < .05) whole‐body crude lipid levels compared to other treatments, but increased moisture levels in the high stocking density.

**TABLE 7:** Final whole‐body proximate composition (% wet weight) of experimental Nile tilapia at different stocking densities for 180 days.

Parameters	Stocking density			
	C‐1 (20 fish/m^3^)	C‐2 (30 fish/m^3^)	C‐3 (40 fish/m^3^)	C‐4 (50 fish/m^3^)
Moisture	70.5 ± 0.06^a^	70.7 ± 1.1^a^	70.2 ± 0.31^a^	70.9 ± 0.04^a^
Protein	20.19 ± 0.11^a^	20. ± 18.121^a^	20.06 ± 1.10^a^	20.02 ± 1.4^a^
Lipid	9.86 ± 1.5^a^	9.88 ± 1.50^a^	9.80 ± 1.7^b^	8.2 ± 1.76^c^
Ash	2.44 ± 0.01^a^	2.46 ± 0.6^b^	1.28 ± 0.3^b^	1.10 ± 0.4^b^

*Note*: Values are the mean ± SD of (*n* = 10). In each row, mean values with different superscripts are significantly different (*p* < .05).

## 4 DISCUSSION

Water quality influences fish biology and physiology, as well as the productivity and well‐being of the rearing system (Bilal et al., 2022; FAO, 2012). Throughout the experimental period, the basic water quality parameters were optimized (Hassan et al., 2022) and therefore, water quality indicators might not be solely responsible for the difference in fish growth seen in this study (Abidin et al., 2022; Yi et al., 1996). Other limiting factors that directly influence are the water quality parameters. The deteriorating quality of the water is linked to the excessive stocking density and suffering fish with high ammonia and organic load which is responsible for the low DO level causing stunted growth and contaminated environment (Person et al., 2008; Romanova et al., 2018). While the water quality of the current study was within an acceptable range that was appropriate for the growth and well‐being of the Nile tilapia, the ideal water quality parameters provided fish with the least amount of stress, which in turn enhanced growth in fish (Bwanika et al., 2007; Ntsama et al., 2018). Wu et al. (2023) suggest that the selective breeding can boost aquaculture productivity (Ponzoni et al., 2008) producing a new strain of Nile tilapia, leading to the maximum growth performance and reducing operational costs and expediting harvesting and (Dey et al., 2000; Haque et al., 2016) enhancing fish survival and production (Abou et al., 2007; Lupatsch et al., 2010). The lower local fish market prices, which will improve affordability, especially for fish farmers and consumers (El‐Saidy & Gaber, 2002). The non‐strain tilapia stocking density is different from that mentioned in the present study (Ntsama et al., 2018). While on the contrary the reduced growth rate of GIFT strain reared under high density was possibly associated with its interactions with the stress due to overcrowding, these factors may increase metabolic demands, consuming more energy to overcome stress compared to growth while higher stocking density may have higher yield but the fish size remains small and takes more time to reach the marketable size (Lupatsch et al., 2010; Wu et al., 2023).

For considering the importance of fish stocking densities in boosting efficiency and production capacity of culture system, many trials have been carried out on different stocking densities of *O. niloticus* using cages as a major culture system in different regions (ecosystem), and variable results in terms of growth performance, survival rate, and portability index were observed (El‐Saidy & Gaber, 2002; Ferdous, Masum, & Ali, 2014; Ferdous, Nahar, et al., 2014). In Pakistan, GIFT *O. niloticus* was introduced and efforts are in progress to obtain the best yield in different culture systems. The present study i.e. determination of the best stocking density that can provide better yield in terms of production and profit in cage culture system is also a continuation of this effort in Pakistan.

There are various problems rising concern about the intensification of fish culture, such as overcrowding stress which in turn exposes the fish to different diseases, and other xenogeneic substances that ultimately influence the growth rate and economic loss to the fish farmers (FAO, 2007; Hassan et al., 2022). The cultural benefits of GIFT strain include better tolerance to variable environmental conditions and feed; moreover, the survival rate is higher than other species. Fish SD is a critical characteristic that influences fish growth and health in a variety of ways (Zhu et al., 2011). The current study of Nile tilapia at different stocking densities ranging from 20 fish/m^3^, 30, 40, and 50 fish/m^3^, we observed a significantly higher weight gain at lower densities such as 20 and 30 fish/m^3^ (Alal, 2018). Increased stocking rates also deteriorated water quality and decreased survival in cement tanks and cage‐farmed fishing have been reported by Zhao et al. (2010) who stocked 400 fish/m^3^ and Lanna et al. (2016) 230.7 fish/m^3^, and Ferdous, Nahar, et al. (2014), and Ferdous, Masum, and Ali (2014) stocked 150 fish individuals/m^3^ that had a negative effect on growth and survival and Huang et al. (2015) stocked 25 fish/m^3^ and obtained maximum growth and production, whereas 230.7 fish/m^3^ were Lanna et al. (2016) and 400 fish/m^3^ were reared by Zhao et al. (2010). The current results were also supported by other investigators (Garcia et al., 2013).

High stocking densities reduce growth and survival rate (Pouey et al., 2011) and influence fish behavior, health, and feed utilization. According to Lin et al. (2004), the fish overpopulation can cause trouble competing for oxygen, food, and space. In such cases, stocking density considers the best ways to maximize the area unit and improve output; however, in other situations, this effect is either temporary or nonexistent (Zhu et al., 2011). Certain fishes can tolerate being overcrowded; however, food competition will limit their growth and result in poor weight gain (Pouey et al., 2011). This might have been the situation in this study when fish stocked at higher quantities grew poorly. According to the findings of this investigation, there was no significant difference in fish survival and growth. Nile tilapia are resilient and can withstand harsh environments, including high SD and will breed even at extremely high densities (Abdel‐Aziz et al., 2021).

The activities of enzymes show the capacity of organisms to estimate the digestion and integration of nutrients in the dietary products (Alal, 2018). The digestive enzyme activities in the fish can help the researchers to evaluate the nutrient's physiological functions helping in resolving the feed compatibility issues for aquaculture (Abolfathi et al., 2012). During the present investigations, we have noticed inverse relations between stocking density as the low stocking density group secreted high concentrations of digestive enzymes, such as cage (1) and cage (2) ultimately favoring the low density in the cage culture.

In the current study, variations in stocking density influenced carcass composition (Table 7). The moisture content was found to be higher under high stocking density. In terms of the impact of SD on overall body growth, the protein was not significantly altered by increased SD. Lipid levels, on the other hand, were considerably lower under high stocking density and similar findings were obtained by Chakraborty et al. (2010) and Lee et al. (2021) who proved that the total fish body content of protein did not change as stocking density increased, however the body content of fat reduced dramatically.

## 5 CONCLUSION

The present research highlighted that the growth, feed consumption, and morphological indices of the Nile tilapia were positively and significantly impacted by the stocking density of 30 fish/m^3^. The findings indicated that Nile tilapia growth decreases as stocking rate increases, and optimal growth performance and survival rate are recorded at low stocking density. The second‐degree polynomial regression indicates that the optimum stocking density for Nile tilapia is 25–35 fish/m^3^ for the sustainable cage culture system. In each culture activity, the ideal stocking density minimizes operating expenses, enhances economic efficiency, and prevents water degradation and enhancing sustainable food production. The findings of this study could be expanded upon in further research since they represent a significant advancement in the development of a Nile tilapia seed‐growing technique that will benefit nursery operators directly. Furthermore, it suggested that achieving better yield, better growth, body composition, survival, stress biomarkers, and digestive enzymes and profit can all be achieved to maximize productivity and profit from Nile tilapia at a stocking rate of 25–35 fish/m^3^ under the cage culture system.

## AUTHOR CONTRIBUTIONS

**Habib Ul Hassan:** Conceptualization (equal); investigation (equal); methodology (equal); writing – original draft (equal). **Amjad Ali:** Supervision (supporting). **Junaid Wattoo:** Conceptualization (equal). **Muhammad Sohail:** Validation (equal). **Muhammad Ahsan Raza:** Data curation (equal). **Syed Adeel Hassan:** Formal analysis (equal). **Roohi Kanwal:** Methodology (equal). **Muhammad Kabir:** Formal analysis (equal). **Naseem Rafiq:** Validation (equal). **Salim Manoharadas:** Writing – review and editing (equal). **Mohammad Rizwan Khan:** Methodology (equal). **Takaomi Arai:** Writing – review and editing (equal).

## FUNDING INFORMATION

This work was supported in part by Universiti Brunei Darussalam under the Faculty/Institute/Centre Research Grant (No. UBD/RSCH/1.4/FICBF(b)/2022/051, UBD/RSCH/1.4/FICBF(b)/2023/057, UBD/RSCH/1.4/FICBF(b)/2023/060).

## CONFLICT OF INTEREST STATEMENT

All authors declare no conflicts of interest.

## Data Availability

The data that support the findings of this study are available on request from the corresponding author.
